# Sample event data on ground beetles (Coleoptera, Carabidae) collected by Biological Station Wijster (BSW) in the years 1959, 1961, 1963, 1965 and 1966

**DOI:** 10.3897/BDJ.13.e171473

**Published:** 2025-11-20

**Authors:** Gijs M. Gerrits, Rikjan Vermeulen, Roel van Klink

**Affiliations:** 1 Wageningen University & Research, Department of Mathematical and Statistical Methods (Biometris), Wageningen, Netherlands Wageningen University & Research, Department of Mathematical and Statistical Methods (Biometris) Wageningen Netherlands; 2 Naturalis Biodiversity Center, Biodiversity Hotspots Group, Leiden, Netherlands Naturalis Biodiversity Center, Biodiversity Hotspots Group Leiden Netherlands; 3 WBBS Foundation, Loon, Netherlands WBBS Foundation Loon Netherlands; 4 German Centre for Integrative Biodiversity Research (iDiv), Leipzig, Germany German Centre for Integrative Biodiversity Research (iDiv) Leipzig Germany; 5 Martin Luther University Halle-Wittenberg, Department of Computer Science, Halle, Germany Martin Luther University Halle-Wittenberg, Department of Computer Science Halle Germany

**Keywords:** arthropods, Dwingelderveld, historical biodiversity data, insect decline, Mantingerbos, pitfall trap, Stichting Willem Bijerink Biologisch Station (WBBS), long-term monitoring, time-series

## Abstract

**Background:**

Historical data on invertebrates are rare and their preservation and FAIR publication are of high importance. Systematically collected historical data can greatly improve the baselines against which trends in biodiversity of these groups are measured. Therefore, we started a data publication project to digitise and publish one of the longest-running insect monitoring schemes in the world.

**New information:**

The raw data presented in the dataset have not been published before. It consists of 45,486 records of 90,070 specimens from 134 species of ground beetles caught between 1959 and 1966 in the Province of Drenthe, the Netherlands. All specimens were identified to species level by taxonomic specialists. The data were collected using a standardised field study set-up with pitfall traps. Extensive cross-checking during digitisation guaranteed an error rate of < 0.1%.

The dataset presented here forms part of a much larger dataset of 300,000 records and close to 1 million individuals of 172 ground beetle species collected in the same area from 1959 to the present day, which will be further digitised in the coming years. The first part of the dataset has been registered in the Global Biodiversity Information Facility (GBIF) and can be found under: https://doi.org/10.15468/3mcqja.

## Introduction

Historical biodiversity data on insects and other invertebrates are rare and often consist of assorted museum specimens. Therefore, publication of historical data obtained from standardised field studies is of high importance, as it can improve our understanding of population and community dynamics in these groups.

This is particularly relevant in the context of the ongoing debate on long-term changes in insect biodiversity. Although several syntheses have documented average declines in insect abundance and diversity, particularly in terrestrial ecosystems (van Klink et al. 2020,van Klink et al. 2023, Turin et al. 2022), this hides a more complex picture. Declines are weaker or absent in freshwater habitats (van Klink et al. 2020, Haase et al. 2023) and vary strongly amongst taxa and regions. Some case studies report stable (Homburg et al. 2019) or mixed trends (Brooks et al. 2012) or even local increases driven by exotic species introductions (Borges et al. 2020). Such heterogeneous outcomes underline that long-term, standardised datasets are essential to disentangle the direction, magnitude and mechanisms of insect community change. The dataset presented here contributes to this debate by providing rare, systematically collected pitfall-trap data on ground beetles (*Carabidae*) from 1959-1966. These data offer historical baselines that can be directly compared with recent surveys to assess long-term trends.

**Figure 1.**
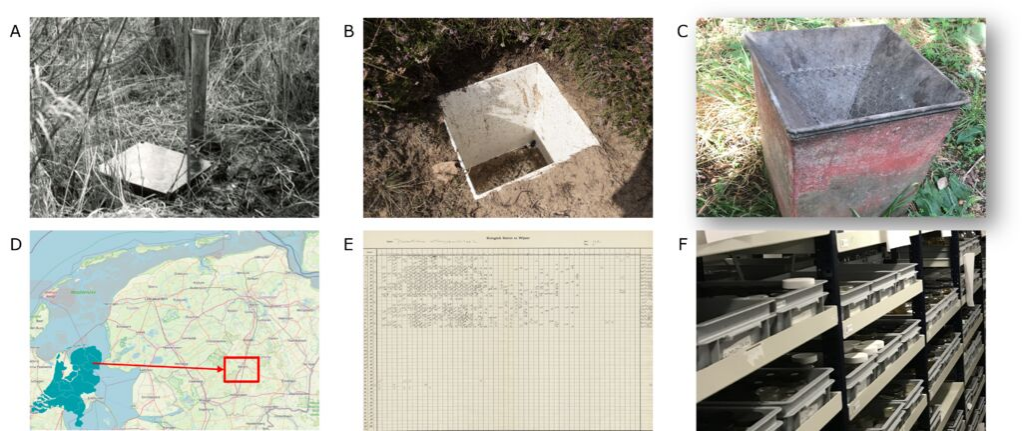
Overview of the pitfall trap design used through the decades (**A-C**), the geographic location of the study area (**D**) as well as an impression of the paper year sheets that make up the paper archive (**E**) and the material stored in the wet collection of Naturalis Biodiversity Center (**F**).

Therefore, we started a project to: (1) bring together, (2) digitise, (3) cross-check and (4) publish a large dataset containing close to 1 million specimens belonging to the family of ground beetles (Coleoptera, Carabidae). The source data consist of a paper archive, lab journals, electronic files in several formats and dry and wet collection material. The data have been collected over the course of 65 years (1959 – present) in the northern Province of Drenthe in the Netherlands. Beetles (and other ground-dwelling invertebrates) were collected using large metal pitfalls dug in flush with the soil surface (Den Boer 1977). Sampling still continues with the same set-up at a selection of locations, making it, to the best of our knowledge, the longest-running standardised monitoring programme for terrestrial insects in the world. Fig. 1 gives an impression of the field set-up and the archive at Naturalis Biodiversity Center in Leiden, Netherlands.

The monitoring programme of the Biological Station Wijster (BSW), then part of Landbouwhogeschool Wageningen, was started to study population dynamics, using ground beetles as a study system (Den Boer 1977). Some of the primary aims were to test paradigms of population regulation and stabilisation (Den Boer and Reddingius 1996) and the importance of dispersal. The research was conducted in various nature reserves in Drenthe Province, where sites were specifically chosen to represent 'stable' and 'unstable' habitats. The focus of the first sampling sites lay on heathlands, forests and bogs. The studied landscape had already become extremely fragmented by agricultural expansion, providing an opportunity to test the probability of extinction of species with differing dispersal capacities (Den Boer 1979).

**Figure 2.**
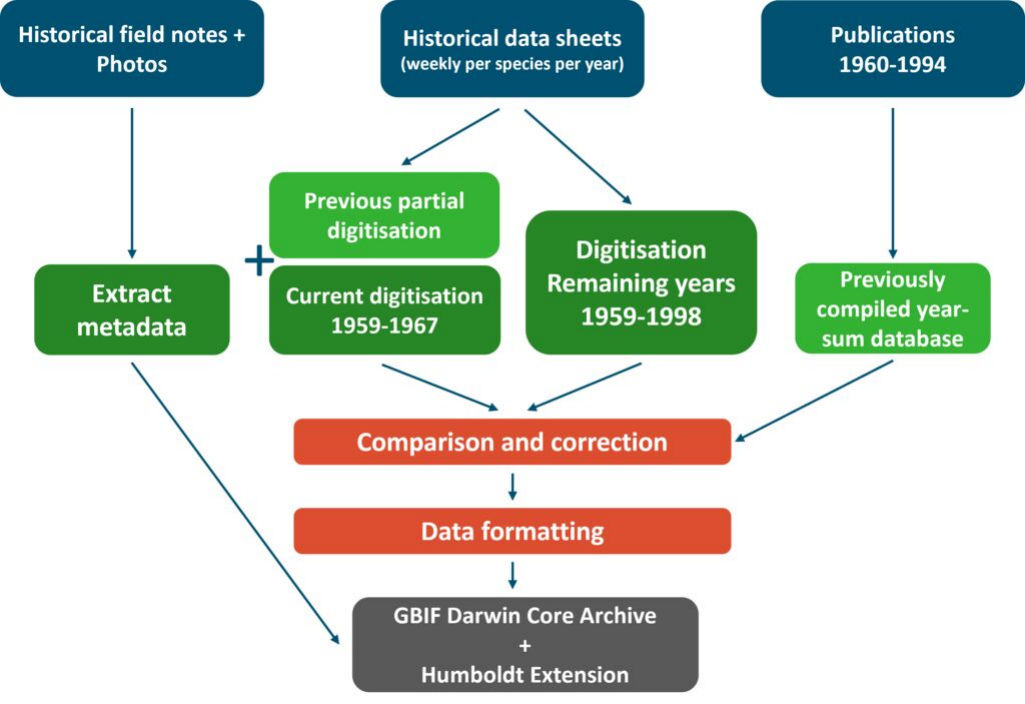
Workflow for the full digitisation of the Biological Station Wijster (BSW) archive. Sources of data and information (blue) lie at the basis of the available data that has been and will be digitised (green), which after extensive checks and corrections is formatted in the Darwin Core format (red) and uploaded as a GBIF Darwin Core archive with Humboldt Extension (grey).

In this datapaper, we present the data of all species caught in a representative selection of years from the first decade of sampling. The goal was to create a stand-alone dataset following rigorous data-checking methods, after which the data would be converted to comply with the Darwin Core standard in order to make it publicly available following FAIR data principles. This first dataset provides the groundwork for digitising the rest of the archive (Fig. 2).

## Project description

### Title

Digitisation and publication of the BSW standard trapping programme

### Personnel

Projectgroup Wijster-data:

- Roel van Klink, German Center for Integrative Biodiversity Research Halle-Jena_Leipzig (iDiv), Martin-Luther University Halle-Wittenberg, Stichting Willem Beijerink Biologisch Station (WBBS Foundation);
- Gijs M. Gerrits, Wageningen University & Research, Naturalis Biodiversity Center.

### Design description

The project entails the digitisation and cross-checking of the first part of the BSW archive (Fig. 2).

### Funding

The project "Pilotproject voor het ontsluiten van data uit het langlopende Wijster loopkeveronderzoek" was funded by NLBIF grant nlbif2023.008.

## Sampling methods

### Sampling description

Ground beetles (Coleoptera, Carabidae) were sampled year-round in square metal pitfall traps with a perimeter of 1 m. The traps were custom-made, based on an earlier design used in the Meijendel coastal dunes (Den Boer 1977, Gerrits and Hemerik 2022). The traps consisted of an outer trap and an inner trap, the latter of which could be lifted out of the outer trap, emptied and placed back with minimal disturbance to the surrounding soil and vegetation. A small hole, covered with fine mesh, provided drainage of rainwater. After 1959, protective covers were placed over the pitfalls to avoid rain, sand and debris building up inside the traps. These were made from opaque metal sheets placed a few centimetres above the trap using pins in the soil.

The traps were placed in groups of three (hereafter referred to as a 'series'), typically set in a straight line with 10 m distance between traps, with the middle trap containing a metal funnel (Fig. 1c), equipped with a container filled with 3% formaldehyde solution as a killing agent. The two outer traps did not contain killing preservative and caught beetles alive. The reason for using these two trap types was because the live traps were supposedly more efficient at catching middle-sized and larger species, whereas the funnel traps caught more smaller species (Den Boer 1977). Generally, the beetles trapped in the live traps were later euthanised and collected for identification.

We obtained the historical locations of the trap series from a range of sources, including the publications (Den Boer and Van Dijk 1994), reports ([Bibr B12789729][Bibr B12789713]), historical maps and field notes from the BSW archive. The coordinates provided in the Darwin Core archive represent the locations to the best of our knowledge, pieced together from the various sources and descriptions. Their accuracy varies between 20 and 10,000 m.

Each sampling year ran from 1 March until 28 (29) February of the next year, with some variation around these dates (see Data Range). The traps were typically emptied weekly, on the same day of the week, but this was variable in winter, with occasionally longer trapping periods around the holidays. All collected specimens were identified to species level, enumerated and, in most cases, separated between males (m) and females (f) by ground-beetle specialists working at the BSW. Species that were not identified as male or female were designated as unknown (u).

### Quality control

Quality control of the digitisation process involved a number of steps and was based on double-entry principles (Barchard and Verenikina 2013). The aim was to achieve a < 0.1% error rate:

1) The archive consists of large paper sheets on which all catches of a species were recorded per trap on each sampling date per year (see Fig. 1e), including marginal row and column sums. We used these marginal sums for comparison with the row and column sums calculated during digitisation. Any differences in marginal totals between our entries and the paper year sheets were then traced to the cell in which the error originated. Both errors in sampling date and trap number could thus be corrected.

The row and column totals on the year sheets were almost perfectly matched with the single entries, showing the high quality of the archive. It should be noted, however, that the year sheets themselves were derived from field and lab notebooks which have mostly been lost. We do, therefore, not know the accuracy of this transcription;

2) The second step was to compare the complete list of species and total capture per species that were digitised with those published by Den Boer (1977);

3) Finally, we compared our data entry of 30 paper sheets (six sheets per year of two rare, two intermediate and two abundant species, with a total of 6326 entries) to a previous digitisation effort of these species and checked each mismatch with the original paper sheets. This showed no errors in our data entry.

## Geographic coverage

### Description

**Figure 3.**
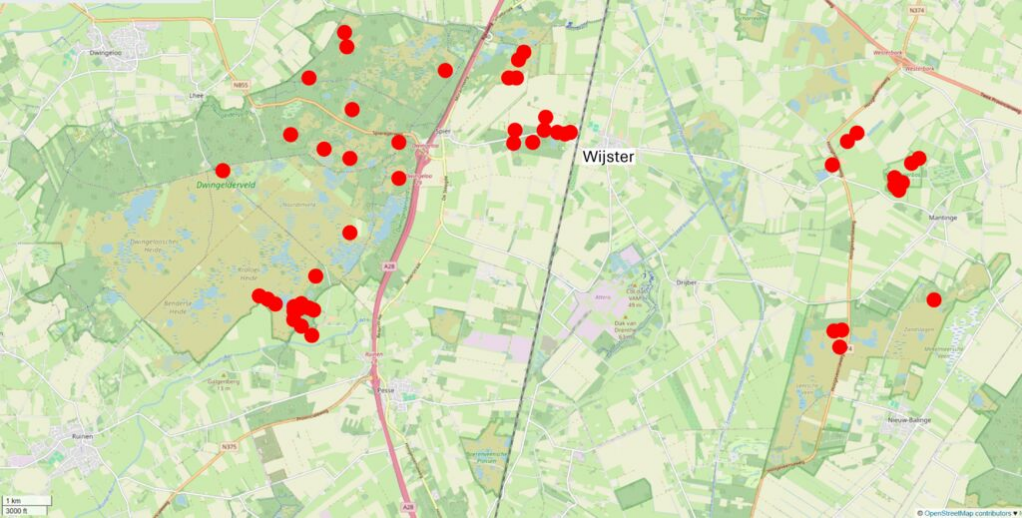
Overview map showing the locations of the pitfall-trapping series included in the BSW dataset (1959–1966). Pitfall series were mostly placed in woodland (darkgreen), wet heathland (sandy green) or open vegetation, such as snippets of dry heathland or inland sand dunes (not shown on map due to small scale or changes since the sampling period). The location of Wijster is also shown, where the Biological Station was based *(Base map © OpenStreetMap contributors)*.

Pitfalls traps were placed in several nature reserves in the Province of Drenthe, in the north of the Netherlands (Fig. 3). We derived the historical locations of the traps from publications (Den Boer and Van Dijk 1994, Verhagen and Vermeulen 2005), historical hand-drawn maps and a report that was written when the biological station was discontinued in 1998 (Van Tol 2000).

### Coordinates

52.7793 and 52.8363 Latitude; 6.4017 and 6.6152 Longitude.

## Taxonomic coverage

### Description

All individuals belonging to the beetle family Carabidae were identified and enumerated. For the complete list of species, see Table 1.

### Taxa included

**Table:** 

Rank	Scientific Name	
kingdom	Animalia	
phylum	Arthropoda	
class	Insecta	
order	Coleoptera	
superfamily	Caraboidea	
family	Carabidae	

## Temporal coverage

**Data range:** 1959-3-07 – 1960-2-24; 1961-2-22 – 1962-2-21; 1963-3-13 – 1964-2-26; 1965-2-24 – 1966-2-23; 1966-2-23 – 1967-2-22.

### Notes

The sampling years in this dataset run from March until February of the next year. The closest sampling dates to 1 March are taken to be the start and end of the running year.

## Collection data

### Collection name

The paper archive of the BSW is now stored in the archive of Naturalis Biodiversity Center, Leiden, the Netherlands. Originally, all samples and bycatch were stored at the BSW in Wijster. When the BSW was dissolved in 1998, part of the samples were stored at Naturalis Biodiversity Center. Of the large amount of original material, it was decided that the catch from all funnel traps was stored for every fourth year (1959 and 1963 in the present dataset). In addition, the catch from the funnel traps was stored for all years for specific locations: B, N, AY and BJ and all material from all traps in other rare habitat types (locations G, L, O, Y, AB, BN, BO, TA, TB, TC, TD, TE and TF). This information is available in the Humboldt extension file.

### Specimen preservation method

ethanol 70%, dry voucher collection.

## Usage licence

### Usage licence

Other

### IP rights notes

CC-BY-4.0

## Data resources

### Data package title

Biological Station Wijster standard trapping programme: Sampling event data for ground beetles (Coleoptera, Carabidae) The data are published as a Darwin Core archive with a Humboldt extension on GBIF. The events follow four hierarchical levels: 1) at the dataset level, the information is stored that is general for the dataset; 2) seriesYear contains the location information for each set of three traps for a whole running year; 3) TrapYear contains the information for each trap for the whole running year: trap type and storage of specimens; 4) trapDate contains the information of each individual sampling event. The occurrences are linked to the lowest level (4). Events during which no ground beetles were collected are present in the dataset with scientificName = Carabidae, individualCount = 0 and occurrenceStatus = 'absent'.

### Resource link

https://doi.org/10.15468/3mcqja

### Alternative identifiers

https://ipt.nlbif.nl/resource?r=bsw_standard_series

### Number of data sets

1

### Data set 1.

#### Data set name

Biological Station WIjster standard trapping programme: Sampling event data for ground beetles (Coleoptera, Carabidae)

**Data set 1.** 

Column label	Column description
basisOfRecord (Occurrence extention)	The specific nature of the data record.
class (Occurrence extention)	The full scientific name of the class in which the dwc:Taxon is classified.
continent (Location Core)	The name of the continent in which the dcterms:Location occurs.
coordinateUncertaintyInMetres (Location Core)	The horizontal distance (in metres) from the given dwc:decimalLatitude and dwc:decimalLongitude describing the smallest circle containing the whole of the dcterms:Location.
country (Location Core)	The name of the country or major administrative unit in which the dcterms:Location occurs.
countryCode (Location Core)	The standard code for the country in which the dcterms:Location occurs.
datasetName (Resource metadata)	The name identifying the dataset from which the record was derived.
day (Event Core)	The verbatim original representation of the date and time information for a dwc:Event.
decimalLatitude (Location Core)	The geographic latitude (in decimal degrees, using the spatial reference system given in dwc:geodeticDatum) of the geographic centre of a dcterms:Location.
decimalLongitude (Location Core)	The ellipsoid, geodetic datum or spatial reference system (SRS), upon which the geographic coordinates given in dwc:decimalLatitude and dwc:decimalLongitude are based.
endDayOfYear (Event Core)	The latest Julian date of the year in which the dwc:Event occurred (1 for 1 January 1, 365 for 31 December, except in a leap year, in which case it is 366).
eventDate (Event Core)	The date-time or interval during which a dwc:Event occurred. For occurrences, this is the date-time when the dwc:Event was recorded.
eventDurationUnit (Humboldt extension)	The units associated with the eco:eventDurationValue.
eventDurationValue (Humboldt extension)	The numeric value for the duration of the dwc:Event.
eventID (Event Core, Occurrence extension)	An identifier for the set of information associated with a dwc:Event (something that occurs at a place and time).
eventRemarks (Event Core)	Comments or notes about the dwc:Event.
eventType (Event Core)	The nature of the dwc:Event.
family (Occurrence extension)	The full scientific name of the family in which the dwc:Taxon is classified.
geodeticDatum (Location Core)	The ellipsoid, geodetic datum or spatial reference system (SRS), upon which the geographic coordinates given in dwc:decimalLatitude and dwc:decimalLongitude are based.
habitat (Event Core)	A category or description of the habitat in which the dwc:Event occurred.
hasMaterialSamples (Humboldt extension)	Material samples were collected during the dwc:Event.
hasNonTargetOrganisms (Humboldt extension)	One or more dwc:Organisms outside the target organismal scopes (eco:targetDegreeOfEstablishmentScope, eco:targetGrowthFormScope and eco:targetLifeStageScope) were detected and reported for this dwc:Event.
hasNonTargetTaxa (Humboldt extension)	One or more dwc:Organisms of taxa outside the target taxonomic scope (the combination of eco:targetTaxonomicScope and eco:excludedTaxonomicScope) were detected and reported for this dwc:Event.
hasVouchers (Humboldt extension)	Specimen vouchers were collected during the dwc:Event.
individualCount (Occurrence extension)	The number of individuals present at the time of the dwc:Occurrence.
isAbsenceReported (Humboldt extension)	Taxonomic absences were reported.
isAbundanceReported (Humboldt extension)	The number of dwc:Organisms collected or observed was reported.
isSamplingEffortReported (Humboldt extension)	The sampling effort associated with the dwc:Event was reported.
kingdom (Occurrence extension)	The full scientific name of the kingdom in which the dwc:Taxon is classified.
latitude (Location Core)	The geographic latitude (in decimal degrees, using the spatial reference system given in dwc:geodeticDatum) of the geographic centre of a dcterms:Location.
locality (Location Core)	The specific description of the place.
locationAccordingTo (Location Core)	Information about the source of this dcterms:Location information.
locationID (Location Core)	An identifier for the set of dcterms:Location information.
longitude (Location Core)	The geographic longitude (in decimal degrees, using the spatial reference system given in dwc:geodeticDatum) of the geographic centre of a dcterms:Location.
materialSampleTypes (Humboldt extension)	A material sample type collected during the dwc:Event.
month (Event Core)	The integer month in which the dwc:Event occurredEvent Core.
occurrenceID (Occurrence extension)	An identifier for the dwc:Occurrence.
occurrenceStatus (Occurrence extension)	A statement about the presence or absence of a dwc:Taxon at a dcterms:Location.
order (Occurrence extension)	The full scientific name of the order in which the dwc:Taxon is classified.
otherCatalogNumbers (Occurrence extension)	A list (concatenated and separated) of previous or alternate fully qualified catalogue numbers or other human-used identifiers for the same dwc:Occurrence, whether in the current or any other dataset or collection.
ownerInstitutionCode (Resource metadata)	The name (or acronym) in use by the institution having ownership of the object(s) or information referred to in the record.
parentEventID (Event Core, Humboldt extension)	An identifier for the broader dwc:Event that groups this and potentially other dwc:Events.
phylum (Occurrence extension)	The full scientific name of the phylum or division in which the dwc:Taxon is classified.
recordedBy (Occurrence extension)	A person, group or organisation responsible for recording the original dwc:Occurrence.
samplingEffort (Event Core)	The amount of effort expended during a dwc:Event.
samplingEffortProtocol (Humboldt extension)	A method or protocol used to determine the sampling effort, denoted by an IRI.
samplingEffortUnit (Humboldt extension)	The units associated with the eco:samplingEffortValue.
samplingEffortValue (Humboldt extension)	The numeric value for the sampling effort expended during the dwc:Event.
samplingPerformedBy (Humboldt extension)	A person, group or organisation responsible for recording the dwc:Event.
samplingProtocol (Event Core)	The names of, references to, or descriptions of the methods or protocols used during a dwc:Event.
sampleSizeUnit (Event Core)	The unit of measurement of the size (time duration, length, area or volume) of a sample in a sampling dwc:Event.
sampleSizeValue (Event Core)	A numeric value for a measurement of the size (time duration, length, area or volume) of a sample in a sampling dwc:Event.
scientificName (Occurrence extension)	The full scientific name, with authorship and date information.
sex (Occurrence extension)	The sex of the biological individual(s) represented in the dwc:Occurrence. M = male, F = female, U = Unknown sex.
siteNestingDescription (Humboldt extension)	Textual description of the hierarchical sampling design.
startDayOfYear	The earliest Julian date of the year in which the dwc:Event occurred (1 for 1 January, 365 for 31 December, except in a leap year, in which case it is 366).
stateProvince (Location Core)	The name of the next smaller administrative region than country (state, province, canton, department, region etc.), in which the dcterms:Location occurs.
targetLifeStageScope (Humboldt extension)	The age classes or life stages of the dwc:Organisms targeted for sampling during the dwc:Event.
targetTaxonomicScope (Humboldt extension)	The taxonomic group targeted for sampling during the dwc:Event.
taxonCompletenessReported (Humboldt extension)	Statement about whether the taxonomic completeness of the dwc:Event was assessed.
taxonRank (Occurrence extension)	The taxonomic rank of the most specific name in the dwc:scientificName.
type (Resource metadata)	The nature or genre of the resource.
verbatimIdentification (Occurrence extension)	A string representing the taxonomic identification as it appeared in the original record.
verbatimLocality (Location Core)	The original textual description of the place.
verbatimSiteNames (Humboldt extension)	A list (concatenated and separated) of original site names.
voucherInstitutions (Humboldt extension)	A list (concatenated and separated) of the names or acronyms of the institutions where vouchers collected during the dwc:Event were deposited.
year (Event Core)	The four-digit year in which the dwc:Event occurred, according to the Common Era Calendar.

## Additional information

As with all datasets, there are a number of issues that should be taken into consideration when using the dataset:

1) The catch from all traps was normally collected weekly, but exceptions were made in winter, where occasionally weeks were skipped or collection days were postponed to avoid holidays. Hence, the catch accumulated until the next collection date. The catch was not collected on the following dates: 6 March 1963, 13 March 1963, 25 December 1963, 24 November 1965, 12 January 1966, 19 January 1966, 26 January 1966, 9 February 1966, 16 February 1966, 30 November 1966, 11 January 1967, 15 February 1967.

2) At present, we have a poor overview of the functionality of individual traps for each event. We know that the traps were affected by rainwater running into the traps, groundwater pushing the traps out of the ground and damage to the traps by livestock or humans, but because this was not systematically recorded, we have not included the sparse information that is available to us in the dataset. Previous analyses have ignored trap functionality and added up all dates and all three traps of a running year for analysis, thereby implicitly assuming such disturbances to be homogeneously distributed amongst traps and years.

3) Some taxonomically close species were not separated during identification, mostly because these pairs were not recognised as separate species at the time of identification (Den Boer pers. comm. 1999, Van Dijk pers. comm. 1999):

- *Calathus melanocephalus* / *C. cinctus* (= *C. erythroderus*);
- *Bradycellus collaris* (= *B. caucasicus*) / *B. harpalinus*;
- *Pterostichus nigrita* / *P. rhaeticus*;
- *Bembidion lampros* / *B. properans* (systematically identified as B. lampros);
- *Poecilus versicolor* (= *Pterostichus coerulescens*) / *P. cupreus*. *P. cupreus* is currently present in the study area, but it is unclear if it was present in the historical period. This species pair was not flagged in 1999 by the original data collectors.

Taxa where additional species may have been overlooked:

- *Agonum fuliginosum* (may have been mixed with *A. gracile* and possibly other *Agonum* species);
- *Asaphidion flavipes* group;
- *Amara*;
- *Harpalus*.

4) Please take note that, in the GBIF occurrence file, "Carabidae" is added as 0 values in the scientificName column. These should not be counted as a species. The total number of species is 134.

## Figures and Tables

**Table 1.** Species list of ground beetles caught in the Wijster Research Programme in the years 1959, 1961, 1963, 1965 and 1966. Scientific names follow the Dutch Species Register - Nederlands Soortenregister (Creuwels and Pieterse 2024).

	**Scientific name**	**Original identification**
1	*Abax parallelepipedus* (Piller & Mitterpacher, 1783)	*Abax ater* Villers
2	*Acupalpus flavicollis* (Sturm, 1825)	*Acupalpus flavicollis* St.
3	*Acupalpus parvulus* (Sturm, 1825)	*Acupalpus dorsalis* F.
4	*Agonum emarginatum* (Gyllenhal, 1827)	*Agonum moestum* Dfts.
5	*Agonum ericeti* (Panzer, 1809)	*Agonum ericeti* Panz.
6	*Agonum fuliginosum* (Panzer, 1809)	*Agonum fuliginosum* Panz.
7	*Agonum gracile* Sturm, 1824	*Agonum gracile* Gyll.
8	*Agonum marginatum* (Linnaeus, 1758)	*Agonum marginatum* L.
9	*Agonum muelleri* (Herbst, 1784)	*Agonum mülleri* Hbst.
10	*Agonum munsteri* (Hellén, 1935)	*Agonum munsteri* Hellén
11	*Agonum sexpunctatum* (Linnaeus, 1758)	*Agonum sexpunctatum* L.
12	*Agonum versutum* Sturm, 1824	*Agonum versutum* Gyll.
13	*Amara aenea* (De Geer, 1774)	*Amara aenea* de Geer
14	*Amara apricaria* (Paykull, 1790)	Amara apricaria Payk.
15	*Amara aulica* (Panzer, 1796)	*Amara aulica* Panz.
16	*Amara bifrons* (Gyllenhal, 1810)	Amara bifrons Gyll.
17	*Amara brunnea* (Gyllenhal, 1810)	*Amara brunnea* Gyll.
18	*Amara communis* (Panzer, 1797)	*Amara communis* Panz.
19	*Amara consularis* (Duftschmid, 1812)	*Amara consularis* Dfts.
20	Amara convexior Stephens, 1828	*Amara convexior* Steph.
21	*Amara equestris* (Duftschmid, 1812)	*Amara equestris* Dfts.
22	*Amara famelica* Zimmermann, 1832	Amara famelica Zimm.
23	*Amara familiaris* (Duftschmid, 1812)	*Amara familiaris* Dfts.
24	*Amara fulva* (Müller, 1776)	*Amara fulva* de Geer
25	*Amara infima* (Gyllenhal, 1812)	*Amara infima* Dfts.
26	*Amara lunicollis* Schiødte, 1837	*Amara lunicollis* Schiödte
27	*Amara makolskii* Roubal, 1923	*Amara pseudocommunis* Burak.
28	*Amara plebeja* (Gyllenhal, 1810)	*Amara plebeja* Gyll.
29	*Amara praetermissa* (Sahlberg, 1827)	*Amara praetermissa* Sahlb.
30	*Amara quenseli* (Schönherr, 1806)	*Amara quenseli* Schönherr
31	*Amara similata* (Gyllenhal, 1810)	Amara similata Gyll.
32	*Amara spreta* Dejean, 1831	*Amara spreta* Dej.
33	*Amara tibialis* (Paykull, 1798)	*Amara tibialis* Payk.
34	*Anchomenus dorsalis* (Pontoppidan, 1763)	*Agonum dorsale* Pontopp.
35	*Anisodactylus binotatus* (Fabricius, 1787)	*Anisodactylus binotatus* F.
36	*Asaphidion flavipes* (Linnaeus, 1760)	*Asaphidion falvipes* L.
37	*Badister bullatus* (Schrank, 1798)	*Badister bipustulatus* F.
38	*Bembidion bruxellense* Wesmael, 1835	*Bembidion rupestre* L.
39	*Bembidion doris* (Panzer, 1796)	*Bembidion doris* Gyll.
40	*Bembidion femoratum* Sturm, 1825	*Bembidion femoratum* St.
41	*Bembidion guttula* (Fabricius, 1792)	*Bembidion guttula* F.
42	*Bembidion humerale* Sturm, 1825	*Bembidion humerale* St.
43	*Bembidion lampros* (Herbst, 1784)	*Bembidion lampros* Hrbst.
44	*Bembidion minimum* (Fabricius, 1792)	*Bembidion minimum* F.
45	*Bembidion nigricorne* Gyllenhal, 1827	*Bembidion nigricorne* Gyll.
46	*Bembidion quadrimaculatum* (Linnaeus, 1760)	*Bembidion quadrimaculatum* L.
47	*Bembidion tetracolum* Say, 1823	*Bembidion ustulatum* L.
48	*Blethisa multipunctata* (Linnaeus, 1758)	*Blethisa multipunctata* L.
49	*Bradycellus caucasicus* (Chaudoir, 1846)	*Bradycellus collaris* Payk.
50	*Bradycellus csikii* Laczó, 1912	*Bradycellus csikii* Laczó
51	*Bradycellus harpalinus* (Audinet-Serville, 1821)	*Bradycellus harpalinus* Dej.
52	*Bradycellus ruficollis* (Stephens, 1828)	*Bradycellus similis* Dej.
53	*Broscus cephalotes* (Linnaeus, 1758)	*Broscus cephalotes* L.
54	*Calathus ambiguus* (Paykull, 1790)	*Calathus ambiguus* Payk.
55	*Calathus erratus* (Sahlberg, 1827)	*Calathus erratus* Sahlb.
56	*Calathus fuscipes* (Goeze, 1777)	*Calathus fuscipes* Goeze
57	*Calathus melanocephalus* (Linnaeus, 1758)	*Calathus melanocephalus* L.
58	*Calathus mollis* (Marsham, 1802)	*Calathus mollis* Mrsh.
59	*Calathus rotundicollis* Dejean, 1828	*Calathus piceus* Marsh.
60	*Calodromius spilotus* (Illiger, 1798)	*Dromius quadrinotatus* Panz.
61	*Carabus arcensis* Herbst, 1784	*Carabus arvensis* Hbst.
62	*Carabus cancellatus* Illiger, 1798	*Carabus cancellatus* Ill.
63	*Carabus granulatus* Linnaeus, 1758	*Carabus granulatus* L.
64	*Carabus nemoralis* Müller, 1764	*Carabus nemoralis* Müll.
65	*Carabus nitens* Linnaeus, 1758	*Carabus nitens* L.
66	*Carabus problematicus* Herbst, 1786	*Carabus problematicus* Hbst.
67	*Chlaenius nigricornis* (Fabricius, 1787)	*Chlaenius nigricornis* F.
68	*Cicindela campestris* Linnaeus, 1758	*Cicindela campestris* L.
69	*Cicindela hybrida* Linnaeus, 1758	*Cicindela hybrida* L.
70	*Cicindela sylvatica* Linnaeus, 1758	*Cicindela silvatica* L.
71	*Clivina fossor* (Linnaeus, 1758)	*Clivina fossor* L.
72	*Cymindis macularis* Fischer von Waldheim, 1824	*Cymindis macularis* Dej.
73	*Cymindis vaporariorum* (Linnaeus, 1758)	*Cymindis vaporariorum* L.
74	*Dromius agilis* (Fabricius, 1787)	*Dromius agilis* F.
75	*Dromius quadrimaculatus* (Linnaeus, 1758)	*Dromius quadrimaculatus* L.
76	*Dyschirius globosus* (Herbst, 1784)	*Dyschirius globosus* Herbst.
77	*Dyschirius politus* (Dejean, 1825)	*Dyschirius politus* Dej.
78	*Dyschirius thoracicus* (Rossi, 1790)	*Dyschirius arenosus* Steph.
79	*Elaphrus cupreus* Duftschmid, 1812	*Elaphrus cupreus* Dfts.
80	*Elaphrus riparius* (Linnaeus, 1758)	*Elaphrus riparius* L.
81	*Epaphius secalis* (Paykull, 1790)	*Trechus secalis* Payk.
82	*Harpalus affinis* (Schrank, 1781)	*Harpalus aeneus* F.
83	*Harpalus anxius* (Duftschmid, 1812)	*Harpalus anxius* Dfts.
84	*Harpalus distinguendus* (Duftschmid, 1812)	*Harpalus distinguendus* Dfts.
85	*Harpalus laevipes* Zetterstedt, 1828	*Harpalus quadripunctatus* Dej.
86	*Harpalus latus* (Linnaeus, 1758)	*Harpalus latus* L.
87	*Harpalus rufipalpis* Sturm, 1818	*Harpalus rufitarsis* Dfts.
88	*Harpalus rufipes* (De Geer, 1774)	*Harpalus pubescens* Müll
89	*Harpalus smaragdinus* (Duftschmid, 1812)	*Harpalus smaragdinus* Dfts.
90	*Harpalus solitaris* Dejean, 1829	*Harpalus fuliginosus* Dfts.
91	*Harpalus tardus* (Panzer, 1796)	*Harpalus tardus* Panz.
92	*Leistus rufomarginatus* (Duftschmid, 1812)	*Leistus rufomarginatus* Dfts.
93	*Leistus spinibarbis* (Fabricius, 1775)	*Leistus spinibarbis* F.
94	*Leistus terminatus* (Panzer, 1793)	*Leistus rufescens* F.
95	*Limodromus assimilis* (Paykull, 1790)	*Agonum assimile* Payk.
96	*Limodromus krynickii* (Sperk, 1835)	*Agonum krynickii* Sperk.
97	*Loricera pilicornis* (Fabricius, 1775)	*Loricera pilicornis* F.
98	*Masoreus wetterhallii* (Gyllenhal, 1813)	*Masoreus wetterhallii* Gyll.
99	*Miscodera arctica* (Paykull, 1798)	*Miscodera arctica* Payk.
100	*Nebria brevicollis* (Fabricius, 1792)	*Nebria brevicollis* F.
101	*Nebria salina* Fairmaire & Laboulbène, 1854	*Nebria salina* Fairm.
102	*Notiophilus aestuans* Dejean, 1826	*Notiophilus pusillus* Waterh.
103	*Notiophilus aquaticus* (Linnaeus, 1758)	*Notiophilus aquaticus* L.
104	*Notiophilus biguttatus* (Fabricius, 1779)	*Notiophilus biguttatus* F.
105	*Notiophilus germinyi* Fauvel, 1863	*Notiophilus germinyi* Fauv.
106	*Notiophilus palustris* (Duftschmid, 1812)	*Notiophilus palustris* Dfts.
107	*Notiophilus rufipes* Curtis, 1829	*Notiophilus rufipes* Curtis
108	*Olisthopus rotundatus* (Paykull, 1790)	*Olisthopus rotundatus* Payk.
109	*Oxypselaphus obscurus* (Herbst, 1784)	*Agonum obscurum* Hbst.
110	*Panagaeus cruxmajor* (Linnaeus, 1758)	*Panageus crux-major* L.
111	*Patrobus atrorufus* (Ström, 1768)	*Patrobus atrorufus* Ström
112	*Philorhizus melanocephalus* (Dejean, 1825)	*Dromius melanocephalus* Dej.
113	*Platynus livens* (Gyllenhal, 1810)	*Agonum livens* Gyll.
114	*Poecilus lepidus* (Leske, 1785)	*Pterostichus lepidus* Leske
115	*Poecilus versicolor* (Sturm, 1824)	*Pterostichus coerulescens* L.
116	*Pterostichus anthracinus* (Panzer, 1795)	*Pterostichus anthracinus* Illig.
117	*Pterostichus diligens* (Sturm, 1824)	*Pterostichus diligens* Sturm.
118	*Pterostichus melanarius* (Illiger, 1798)	*Pterostichus vulgaris* L.
119	*Pterostichus minor* (Gyllenhal, 1827)	*Pterostichus minor* Gyll.
120	*Pterostichus niger* (Schaller, 1783)	*Pterostichus niger* Schall.
121	*Pterostichus nigrita* (Paykull, 1790)	*Pterostichus nigrita* F.
122	*Pterostichus oblongopunctatus* (Fabricius, 1787)	*Pterostichus oblongopunctatus* F.
123	*Pterostichus quadrifoveolatus* Letzner, 1852	*Pterostichus angustatus* Dfts.
124	*Pterostichus strenuus* (Panzer, 1796)	*Pterostichus strenuus* Panz.
125	*Pterostichus vernalis* (Panzer, 1796)	*Pterostichus vernalis* Panz.
126	*Stenolophus mixtus* (Herbst, 1784)	*Stenolophus mixtus* Hbst.
127	*Stomis pumicatus* (Panzer, 1796)	*Stomis pumicatus* Panz.
128	*Syntomus foveatus* (Geoffroy, 1785)	*Metabletus foveatus* Fourer.
129	*Syntomus truncatellus* (Linnaeus, 1760)	*Metabletus truncatellus* L.
130	*Synuchus vivalis* (Illiger, 1798)	*Synuchus nivalis* Panz.
131	*Trechus obtusus* Erichson, 1837	*Trechus obtusus* Er.
132	*Trechus quadristriatus* (Schrank, 1781)	*Trechus quadristriatus* Schrk.
133	*Trichocellus cognatus* (Gyllenhal, 1827)	*Trichocellus cognatus* Gyll.
134	*Trichocellus placidus* (Gyllenhal, 1827)	*Trichocellus placidus* Gyll.
